# Intestinal Fatty Acid Binding Protein Levels in Pediatric Celiac Patients in Transition From Active Disease to Clinical and Serological Remission

**DOI:** 10.1097/PG9.0000000000000070

**Published:** 2021-03-30

**Authors:** Assaf Hoofien, Anat Guz-Mark, Noam Zevit, Tsachi Tsadok Perets, Amit Assa, Olga Layfer, Manar Matar, Vered Nachmias-Friedler, Ari Silbermintz, Raanan Shamir

**Affiliations:** From the * Institute of Gastroenterology, Nutrition and Liver Diseases, Schneider Children’s Medical Center, Petach Tikva, Israel; † Sackler Faculty of Medicine, Tel Aviv University, Tel Aviv, Israel; ‡ Gastroenterology Laboratory, Rabin Medical Center—Beilinson Hospital, Peath Tikva, Israel; § Adelson School of Medicine, Ariel University, Ariel, Israel; ‖ Faculty of Health Sciences, Ben-Gurion University of the Negev, Beer-Sheba, Israel; 6Pediatric Gastroenterology and Nutrition Unit, Lady Davis Carmel Medical Center, Haifa, Israel.

**Keywords:** noninvasive diagnosis, follow-up, celiac, children, markers, enteropathy

## Abstract

Celiac disease (CD) is increasingly diagnosed without endoscopy. As such, the need for accurate serological markers to aid in the diagnosis and follow-up of CD has increased. Intestinal fatty acid binding protein (I-FABP) is a cytosolic protein present in enterocytes, whose blood levels reflect mucosal damage in a reliable and quantifiable way. The aim of this study was to compare I-FABP levels in newly diagnosed patients with CD and to examine changes in levels following 6 months of gluten-free diet (GFD).

**Methods:**

A prospective observational case control study of pediatric patients diagnosed with CD, with measurements of tissue transglutaminase IgA (TTG-IgA) and I-FABP levels at diagnosis and after 6 months of gluten free diet were compared to a control group of nonceliac patients.

**Results:**

This study included 35 patients and 32 controls. The CD group had higher I-FABP levels at diagnosis compared with the control group (median 641.7 pg/mL versus 334 pg/mL; *P* < 0.05). I-FABP levels significantly differed between patients presenting with TTG-IgA level 3–10 times the upper limit of normal (ULN) compared with those presenting with values >10 times ULN (median 432.2 pg/mL versus 796.2 pg/mL; *P* < 0.05). Patients with CD had a significant decrease in median I-FABP levels after 6 months of GFD (median 268.2 pg/mL), paralleling a decrease in TTG-IgA and GFD adherence.

**Conclusions:**

I-FABP levels are increased in patients with CD at diagnosis compared with controls and decrease significantly while patients adhere to GFD.

**What Is Known**

- Follow-up of patients with celiac disease (CD) requires surveillance of serological markers, but these do not directly reflect tissue inflammation and damage.
- Intestinal fatty acid binding protein (I-FABP) is a reliable marker of intestinal mucosal damage, and its resolution.

**What Is New**

- I-FABP can discriminate patients with CD from controls, and its level further accords with elevation of other markers.
- I-FABP levels decrease significantly during long-term follow-up of patients with CD with good adherence to a gluten-free diet.

## INTRODUCTION

Celiac disease (CD) is an immune-mediated enteropathy characterized by gluten-induced small intestinal damage in genetically susceptible individuals (1). Until recently, the diagnosis of CD was based on compatible duodenal biopsies in patients with elevated serological markers (such as anti-tissue transglutaminase [TTG-IgA], and anti-endomysial [anti-EMA] antibodies). In 2012, the European Society for Pediatric Gastroenterology, Hepatology and Nutrition (ESPGHAN) revised CD diagnostic criteria, allowing for nonendoscopy based diagnoses of CD in pediatric patients with highly elevated levels (>10 times upper normal limits) of TTG-IgA, a positive anti-EMA on a separate occasion and a positive HLA DQ2 or DQ8. In 2020, these criteria were expanded to include asymptomatic patients as well, and the requirement for HLA testing was removed. However, patients not meeting these minimum criteria for noninvasive diagnosis still require endoscopy with duodenal biopsies to diagnose CD (1).

The treatment for CD is a strict gluten-free diet (GFD) for life. Clinical and serological follow-up is recommended following initiation of treatment and longitudinally after diagnosis because serological markers are correlated with GFD adherence (2). However, studies have demonstrated that duodenal inflammation persists despite clinical and serological improvement in some patients (3). In cases where there is no clear clinical or serological remission, repeat endoscopy with duodenal biopsies may be considered, to assess for mucosal healing (4). Thus, despite the usefulness of the currently available serological markers for both diagnosis and follow-up of patients with CD, their sensitivity and specificity remain limited as demonstrated in a recent meta-analysis demonstrating the poor performance of celiac serology in identifying persistent villous atrophy in patients with CD (5).

Intestinal fatty acid-binding protein (I-FABP) facilitates intercellular metabolism and transport of long-chain fatty acids. It is a small, unbound, cytosolic protein present in enterocytes and is highly concentrated at the tips of the jejunal villi. It is rapidly released into the systemic circulation during cell damage (6,7). Elevated levels of I-FABP have been identified in blood and urine samples of patients with a wide variety of intestinal pathologies, including mesenteric thrombosis, necrotizing enterocolitis, and Crohn’s disease (8–10). Clinical research in patients with CD has shown that I-FABP may have a role in both the noninvasive diagnosis and follow-up of CD since serum I-FABP levels tend to reflect mucosal damage in a more reliable and quantifiable way than other available markers and has been shown to elevate and normalize faster than other markers (11,12).

Retrospective studies have shown a significant correlation between I-FABP serum levels in pediatric patients with CD and histological Marsh scores at diagnosis (13). A prospective study has suggested that I-FABP may have a role in the follow-up of patients with CD (14).

The aim of this study was to prospectively assess the value of I-FABP in the diagnosis and follow-up of pediatric CD compared with standard serological follow-up.

## METHODS

### Subjects

This study was a prospective longitudinal cohort study with a control group. The study included children younger than 17 years of age undergoing endoscopy for suspected CD at Schneider Children’s Medical Center of Israel (SCMCI). Inclusion criteria included clinical suspicion of CD (as per ESPGHAN 2020 guidelines for the diagnosis of CD^1^) and elevated celiac serology (defined as ≥3 times upper limit of normal [ULN] value of TTG-IgA). The diagnosis was confirmed by duodenal biopsy results compatible with CD (Marsh score ≥2). Following diagnosis, all patients received dietary consultation and training for GFD by a pediatric dietician with experience with CD.

The control group included children younger than 17 years of age, undergoing endoscopy for investigation of nonspecific abdominal pain, with negative celiac serology, and no other preexisting gastrointestinal disease. Patients with duodenal biopsies not compatible with Marsh grade 0 were excluded.

Additional exclusion criteria for both groups included conditions previously reported to induce elevations of I-FABP (inflammatory bowel disease, bowel ischemia, recurrent bowel obstruction, primary biliary cholangitis, bile duct obstruction, hepatic failure, hepatic malignancy) (15); following acute gastrointestinal injury (surgical or traumatic) in the last 3 months; chronic or acute use of nonsteroidal anti-inflammatory drugs in the week preceding endoscopy (16); intense physical activity (defined as >1 hour/d) in the 2 days before the endoscopy (17); or trisomy 21.

The study was conducted according to the Declaration of Helsinki and approved by the institutional review board of Rabin Medical Center.

### Study Protocol

Following signed informed consent, demographic and clinical data were collected. Data included age, ethnicity, gender, weight, height, age at symptom appearance, recent celiac serology, complete blood count (CBC), iron and ferritin levels, and liver enzyme serum levels. A serum sample for I-FABP was obtained at the time of the diagnostic endoscopy, before the insertion of the endoscope, via a peripheral IV cannula placed during the anesthesia protocol. Samples were centrifuged and serum was frozen at −20°C until batch analysis was performed. I-FABP concentrations in patient samples was determined using a Quantikine ELISA Human FABP2/I-FABP Immunoassay kit (R&D Systems Inc., Minneapolis, MN) according to the manufacturer’s instructions.

For the CD group, I-FABP concentrations were also measured 6 months after the initiation of a GFD. Clinical symptoms were assessed, weight and height measured, and GFD adherence was assessed (using the modified Biagi questionnaire (18)). In addition, routine CD follow-up laboratory tests were drawn (CD serology, CBC, liver enzyme assays, ferritin).

Because TTG-IgA from various centers had been used for patient referrals, the results are recorded as times above ULN.

### Statistical Analysis

For descriptive purposes, plasma I-FABP levels are presented as median and ranges. Pearson correlation was used to assess the relationship between IFABP and age. We calculated the area under each curve (AUC or c-statistic) to assess the criterion-validity of the tested in diagnosing CD. AUC larger than 0.8 indicate large effect sizes (19). A *p* value below 0.05 was considered statistically significant. All analyses were conducted using IBM-SPSS version 25 (IBM-SPSS, Armrok, NY).

## RESULTS

Thirty-five patients with elevated TTG-IgA serology, and histological confirmation of CD, were included in the study group (10 patients with Marsh scores 3B and 25 with Marsh 3C); and 32 patients were included in the control group.

Gender did not differ between groups (males 21.8% and 31.4% in the control and study groups, respectively, *P* = 0.4), nor did other reported chronic medical diagnoses (that have no known reported effect on serum I-FABP levels), including asthma, diabetes mellitus, eosinophilic esophagitis, hypothyroidism, and thalassemia minor (9% versus 11%, *P* = 1). Patients with CD were younger at the time of inclusion (mean CD 7.8 ± 3 years versus control 12 ± 4 years, *P* < 0.05). Furthermore, less *H. pylori* was found in gastric biopsies of patients with CD (CD 8.5% versus 32.3% in controls, *P* < 0.05).

**FIGURE 1.**
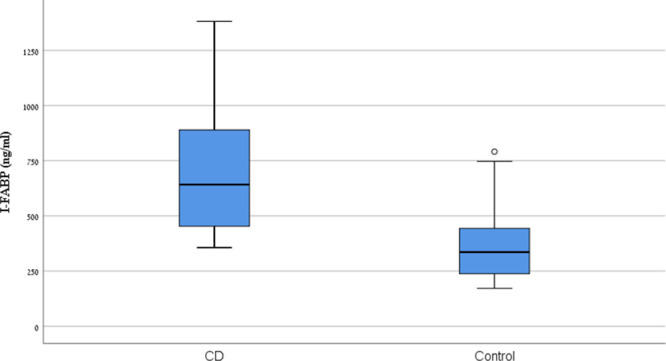
I-FABP levels at time of endoscopy, study group vs. control group. CD = celiac disease.

At the time of endoscopy, the median TTG-IgA level in patients with CD was 19.3 times ULN (range 3.8–111.1). I-FABP levels in patients with CD were significantly higher than those of the controls (CD median 641.7 pg/mL, range 356.2–1381 versus control median 334 pg/mL, range 171.43–791, *P* < 0.05) (Fig. 1).

In the study group, 22.8% of patients had TTG-IgA < 10 times ULN. The median value of I-FABP for this sub-group with was 432.2 pg/mL (range 3.8–8, *P* < 0.05 in comparison to control group). Patients with TTG-IgA > 10 times ULN, median I-FABP was significantly higher at 796.2 pg/mL (range 10.2–111.1, *P* < 0.05 in comparison to control group).

There was a weak but significant negative correlation between I-FABP levels and age at endoscopy (Spearman’s rho −0.46, *P* < 0.05).

I-FABP concentrations did not differ significantly between H. *Pylori* positive (34.3%, 11/32) and negative (68.7%, 22/32) control patients (as determined by histology) regardless of the presence of gastritis (median: HP+ 271.6 pg/mL versus HP− 365.4 pg/mL; *P* = 0.17).

**FIGURE 2.**
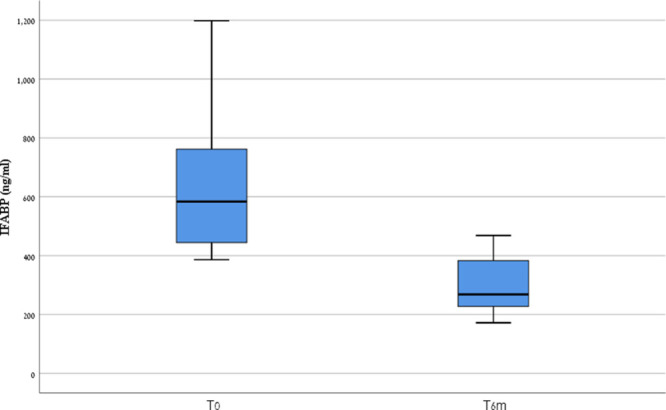
I-FABP levels at diagnosis (T0) of celiac disease and at 6-month follow-up (T6m).

Samples were available from 33% (13/36) of patients with CD following 6 months of GFD. Biagi questionnaires were collected from 92% (12/13) of them and demonstrated high levels of adherence to GFD (median score of 4). TTG-IgA levels declined significantly over 6 months follow-up (median 2 times ULN, range 0.3–13.7 times ULN). Eighty-five percent (11/13) of patients reported being asymptomatic. In patients with CD, I-FABP levels decreased significantly while on GFD (median 268.2 pg/mL versus 641.7 pg/mL at diagnosis, range 171–468, *P* < 0.05) (Fig. 2).

## DISCUSSION

In this study, we showed that in pediatric patients with CD, I-FABP levels were significantly elevated at diagnosis and differed from nonceliac patients with abdominal pain. Moreover, I-FABP levels were reduced significantly following 6 months of good adherence to GFD. These findings recapitulate both the results of a study examining I-FABP levels in adult CD (12) and pediatric studies that prospectively followed patients from diagnosis to treatment over shorter or equivalent periods of time (13,14).

We further demonstrated that I-FABP levels at diagnosis differ between patients with CD presenting with moderate range TTG-IgA elevations (3–10 times ULN) and those with highly elevated TTG-IgA (>10 times ULN). Adrinaase *et al.* reported similar findings in adults; however, their results were not quantified (14). We believe this is an important observation for future research when assessing I-FABP as a non-invasive diagnostic tool in conjunction with other established tests.

There was no difference in I-FABP levels between children with and without H. *Pylori* in gastric biopsy. This is similar to findings of an adult cohort (20), and could be attributed to the lack of effect of *H. Pylori* (whether by its presence alone or through infection-related gastritis) on I-FABP levels.

Our cohort showed an over-all good response to GFD, as seen from a high adherence questionnaire score, and a significant decrease in TTG-IgA over a 6 month period. Median I-FABP levels were also reduced significantly to a level lower than the median of our control group. Previous work by Adrinaase et al has shown that adult patients with CD, even while under strict GFD and proven normalization of serological markers, still had an elevated I-FABP level, higher than the control group of that cohort (21). This phenomenon was hypothesized to originate from several causes, one of which was age-dependent differences in long-term GFD adherence and mucosal recovery (22). This hypothesis is in conjunction with our cohort being a pediatric cohort of newly diagnosed patients with CD.

Lack of mucosal healing and continued villous atrophy despite adherence to GFD and normalization of serological markers, has been shown to exist in both adult and pediatric cohorts (5,23,24). Since I-FABP is a direct marker of enterocyte injury, it may have a role in complementing other forms of follow-up in patients with CD. Our study validates previous work and shows that I-FABP has a discriminatory role both in the diagnosis and in follow-up of these patients.

Our study has several limitations that must be acknowledged: the lack of repeated biopsies after GFD initiation limits the ability to directly correlate I-FABP levels at follow-up with histological changes. In addition, loss to follow-up of patients with CD at the 6 month follow-up limits the statistical power of our findings.

In conclusion, I-FABP has been established as a sensitive marker of mucosal injury in CD. This prospective controlled study shows that I-FABP levels are significantly elevated in untreated cases of pediatric CD, with a correlation to serological markers. Furthermore, GFD adherence results in significant reduction in serum I-FABP levels, and in children, at 6 months of treatment, reach a level equivalent to non-CD controls. This study adds to the body of research regarding the possible application of I-FABP in the diagnosis and follow-up of patients with CD, enabling its routine use in the future as an established marker of disease activity.
